# A Correlation Between Left Ventricular Systolic Dysfunction, Identified on Global Longitudinal Strain, and Inducible Ischemia on 2D Stress Echocardiography in Type 2 Diabetes With Preserved Ejection Fraction

**DOI:** 10.7759/cureus.79429

**Published:** 2025-02-21

**Authors:** Vinay Kumar Sharma, Rakesh Kumar Tikadar, Sunil K Tripathi, Mohit Tondon, Puneet Aggarwal

**Affiliations:** 1 Cardiology, Fortis Escorts Heart Institute and Research Centre, Delhi, IND; 2 Cardiology, Shyam Shah Medical College, Rewa, IND; 3 Cardiology, Atal Bihari Vajpayee Institute of Medical Sciences and Dr Ram Manohar Lohia Hospital, Delhi, IND

**Keywords:** diabetes mellitus, echocardiography, global longitudinal strain, glycated haemoglobin, stress, ventricular dysfunction

## Abstract

Background: The relationship between glycemic control and cardiovascular outcomes is significant. Subclinical systolic impairment could be the first indicator of diabetic cardiomyopathy, even before detectable changes in ejection fraction.

Aim: To determine the prevalence of left ventricular (LV) systolic dysfunction by global longitudinal strain (GLS) in patients with asymptomatic type 2 diabetes mellitus (T2DM) patients with preserved ejection fraction and correlate GLS with stress echocardiography positivity rates in these patients.

Methods*:* This prospective, observational, cross-sectional, single-center study included 150 asymptomatic T2DM patients with preserved left ventricular ejection fraction (LVEF≥50%). Patients underwent comprehensive echocardiography, which included GLS measurement and stress echocardiography. Patients were categorized based on GLS values (normal:≥-17%; reduced:<-17%) and stress echocardiography results.

Results: The LV systolic dysfunction, defined by reduced GLS, was observed in 37.7% of patients. Patients with reduced GLS were older (64.42±11.01 vs. 55.09±12.20 years, p<0.001), had higher HbA1c levels (8.6±0.99% vs. 7.05±1.05%, p<0.001), and longer diabetes duration (10.43±5.59 vs. 6.32±5.90 years, p<0.001). Stress echocardiography positivity was significantly higher in the reduced GLS group compared to the normal GLS group (17.9% vs 4.3%, p=0.006). Patients with positive stress echocardiography results showed significantly lower average GLS compared to those with negative results (16.16±3.96% vs 18.69±3.45%, p=0.04).

Conclusions: In asymptomatic T2DM patients with preserved LVEF, reduced GLS is associated with a higher rate of positive stress echocardiography results. The LV systolic dysfunction, indicated by reduced GLS, is common in diabetic patients and associated with higher HbA1c and longer diabetes duration.

## Introduction

Diabetes mellitus (DM) has become a major global healthcare problem, causing more than five million deaths worldwide, with a mortality rate of 82.4 per 1,00,000 [1]. DM is associated with high-risk coronary artery disease (CAD) and can also lead to cardiomyopathy [2]. The relationship between glycemic control and cardiovascular outcomes is significant, with literature stating that each 1% reduction in HbA1c correlates with a 16% decrease in the risk of heart failure [3].

The initial functional changes in diabetic cardiomyopathy remain controversial. While left ventricular (LV) diastolic dysfunction has long been considered the earliest alteration, recent evidence suggests that LV systolic dysfunction may precede it [4]. This emerging view proposes that subclinical systolic impairment could be the first indicator of diabetic cardiomyopathy, even before detectable changes in ejection fraction [5,6]. This shift challenges traditional understanding and highlights the need for further research into the early cardiac functional changes in diabetes. Advancements in myocardial function evaluation have led to the development of novel diagnostic techniques. Global longitudinal strain (GLS) has emerged as an effective tool for assessing LV systolic function, proving to be a sensitive indicator of subtle LV myocardial changes [7]. However, LV function may sometimes appear normal at rest, necessitating stress echocardiography to evaluate LV systolic and diastolic reserve during exercise. This approach, combined with speckle-tracking imaging, is particularly valuable in asymptomatic diabetic patients [8]. Recent literature suggests that during stress testing, GLS can aid in identifying ischemic regions by revealing reduced strain in affected segments. By incorporating GLS into stress echocardiography protocols, clinicians can achieve a more comprehensive and accurate assessment of myocardial function [9].

Despite these advancements, a crucial question remains unanswered: what is the correlation between LV systolic dysfunction and inducible ischemia in patients with diabetes? To bridge this knowledge gap, our study aims to assess the prevalence of subclinical LV systolic dysfunction using GLS in asymptomatic T2DM patients with preserved ejection fraction and examine its correlation with stress echocardiography findings of inducible ischemia. Additionally, it seeks to evaluate the impact of normal or reduced GLS on the positivity rate of stress echocardiography, indicating inducible ischemia in these patients.

## Materials and methods

Study design and population

This prospective, observational, cross-sectional, single-center study was conducted at a tertiary care center in India between October 2022 and June 2023. Patients aged above 18 years with type 2 diabetes with preserved LVEF were included in the study. Patients who underwent 2D echocardiography and were later referred for stress echocardiography were included in the study. Exclusion criteria comprised patients with acute coronary syndrome, a history of heart disease, symptomatic heart failure, severe valvular lesions, LVEF <50%, arrhythmia, congenital heart disease, prior cardiac surgery, uncontrolled hypertension, renal insufficiency, liver dysfunction, or lung disease. A total of 150 patients meeting the aforementioned inclusion criteria were enrolled in the study. These participants were subsequently categorized into two groups based on their individual left ventricular global longitudinal strain (LV-GLS) measurements and stress echocardiography outcomes. The study was approved by the Institutional Ethics Committee of Fortis Escort Heart Institute (IEC/2022/OAS/07), and patient consent was signed by the patients before enrollment in the study.

Echocardiography recording and LV-GLS analysis

Transthoracic echocardiography was conducted on all patients at rest using an ultrasound system (GE Vivid E95 USA). LV end-diastolic volume index (EDVI) and end-systolic volume index (ESVI) were determined using Simpson’s biplane method of discs and normalized for body surface area. LVEF was computed and presented as a percentage. LV mass was indexed to body surface area, taking obesity into account. The early diastolic (E) and atrial wave velocities, along with E-wave deceleration time, were measured using pulsed-wave Doppler. The early diastolic velocity (E’) was obtained from the septal mitral annulus, and the E/E’ ratio was calculated to estimate LV filling pressure. Offline analysis of speckle-tracking echocardiography was conducted using GE Vivid E95 (USA) software. The LV-GLS was measured and recorded. In accordance with established literature, LV-GLS value <-17 was classified as impaired, while values ≥-17 were considered normal [10-12].

Stress echocardiography

Stress echocardiography was performed following the American Society of Echocardiography guidelines [13]. Standard views for assessing regional wall motion and thickening included parasternal long and short-axis images, as well as apical four and two-chamber views.

Exercise stress echocardiography

All patients underwent a symptom-limited treadmill exercise test using the standard Bruce protocol. Participants were instructed to exercise to the point of exhaustion. Throughout the examination, blood pressure measurements and 12-lead electrocardiogram recordings were obtained at rest, at two-minute intervals during exercise, at peak exertion, and post-exercise. The test was terminated upon meeting any of the following criteria: emergence of symptoms, arrhythmia, blood pressure abnormalities, or significant ST-segment change.

Pharmacological stress echocardiography

Dobutamine stress echocardiography (DES) was performed for myocardial ischemia assessment. Dobutamine was administered in graded doses (5-40 ug/kg/min) at three-minute intervals. Atropine (0.25-0.50 mg) was given if the target heart rate wasn’t achieved. 12-lead electrogram (ECG) and blood pressure were continuously monitored. The 2D echocardiography images were digitally recorded at rest, low dose, peak stress, and recovery stages. Test endpoints included target heart rate achievement, hypotension, wall motion abnormalities, arrhythmia, severe hypertension, or intolerance symptoms. Stress-induced new or worsening left ventricular wall motion abnormalities were considered positive results. Regional wall motion abnormalities were assessed using a 17-segment, four-point scale model. Maximum metabolic equivalents (METS), heart rate, and blood pressure were recorded.

Sample size calculation

A total of 150 patients meeting the predefined inclusion criteria were enrolled in the study. These participants were subsequently categorized into two groups based on their individual GLS measurements and stress echocardiography outcomes.

$N = \frac{(r + 1) (Z_{\alpha/2} + Z_{1-\beta})^2 \sigma^2}{r d^2}$

 where,

Z = normal deviation at a level of significance (Z is 1.96 for a 5% level of significance)

Z_1_ = normal deviation at 1-β% power with β% of type II error

Z_α/2_​ = Z-score corresponding to the desired significance level (α)

r = n_1_/n_2_ is the ratio of sample size required for two groups

σ = pooled standard deviation

d = difference of means of 2 groups

Statistical analysis

Statistical analysis was performed using SPSS (IBM SPSS Statistics for Windows, Version 25.0. Armonk, NY: IBM Corp; 2017). Continuous variables were expressed as mean±standard deviation, while categorical variables were presented as numbers and percentages. Participants were stratified into two groups based on the test results. For continuous variables, an independent t-test was used for analysis, and for categorical variables, a chi-square test was used. A p-value<0.05 was considered statistically significant.

## Results

Baseline and demographic details

A total of 150 asymptomatic T2DM patients with preserved LVEF were included in the study. Male predominance was observed in the study. The patients were divided into two groups based on their GLS values: normal GLS (≥-17%, n=94) and reduced GLS (<-17%, n=56). Patients with reduced GLS were older than those with normal GLS (64.42±11.01 years vs. 55.09±12.20 years; p=0.000). The mean body mass index (BMI) was slightly higher in the reduced GLS group (25.7±3.62 kg/m2) compared to the normal GLS group (24.7±3.44 kg/m2), but this difference was not statistically significant (p=0.09). Glycaemic control, as measured by HbA1c, was significantly poorer in the reduced GLS group (8.6±0.99% vs. 7.05±1.05%; p=0.000). The duration of diabetes was also significantly longer in patients with reduced GLS (10.43±5.59 years vs. 6.32±5.90 years; p<0.0001). A comparison of demographics based on GLS values is summarized in Table 1.

**Table 1:** Comparison of demographic details among normal and reduced GLS. The data is represented in mean±SD or n (%). p-value <0.05 is considered as statistically significant. BMI: body mass index; DM: diabetes mellitus; GLS: global longitudinal strain; HBA1c: glycated hemoglobin.

Variables	Normal GLS (≥ -17%) (N=94 patients)	Reduced GLS (< -17%) (N=56 patients)	t or chi-square value	p-value
Age, years	55.09 ± 12.20	64.42 ± 11.01	-4.672	<0.001
BMI, kg/m^2^	24.7 ± 3.44	25.7 ± 3.62	-1.674	0.09
HbA1c, %	7.05 ± 1.05	8.6 ± 0.99	-9.292	<0.000
Duration of DM	6.32 ± 5.90	10.43 ± 5.59	-4.209	<0.001
Hypertension	21 (22.3)	24 (42.9)	7.034	0.008
Dyslipidemia	41 (43.6)	33 (58.9)	3.292	0.07
Smoking	19 (20.2)	18 (32.1)	2.688	0.101
Hypothyroidism	23 (24.5)	14 (25.0)	0.005	0.942

2D and stress echocardiography among normal and reduced GLS

Table 2 compares GLS and stress test parameters in asymptomatic diabetic patients with normal versus reduced GLS. No significant differences were observed between the groups in terms of LV mass indexed to body surface area (91.8±12.09 g/m^2^ vs. 93.3±9.50 g/m^2^; p=0.397). Regarding diastolic function parameters, the E wave velocity was significantly lower in the reduced GLS group (0.65±0.11 m/sec vs. 0.70±0.10 m/sec; p=0.005). During stress testing, there were no significant differences in METS achieved (8.57±1.84 vs. 9.11±1.28; p=0.266) between the reduced and normal GLS groups, respectively. The stress echocardiography results showed a significantly higher rate of positive tests in the reduced GLS group compared to the normal GLS group (17.9% vs. 4.3%; p=0.006).

**Table 2:** Comparison of 2D-stress echocardiography parameters among normal and reduced GLS. The data is represented in mean±SD or n (%). A p-value <0.05 is considered statistically significant. BSA: body surface area; GLS: global longitudinal strain; LAVI: left atrial volume index; LVEDVI: left ventricular end-diastolic volume index; LVEF: left ventricular ejection fraction; LVESVI: left ventricular end-systolic volume index.

Variables	Normal GLS (≥ -17%) (N=94 patients)	Reduced GLS (< -17%) (N=56 patients)	t or chi-square value	p-value
Average GLS, %	20.71 ± 2.33	14.89 ± 1.91	15.716	<0.001
LVEF, %	57.9 ± 2.90	56.8 ± 2.63	4.010	<0.001
LV mass / BSA, (g/m^2^)	93.3 ± 9.50	91.8 ± 12.09	0.849	0.397
LVESV1, (ml/m^2^)	19.5 ± 2.73	19.3 ± 2.70	0.334	0.739
LVEDV1, (ml/m^2^)	41.7 ± 8.60	43.5 ± 6.70	-1.308	0.19
LAVI, (ml/m^2^)	26.44 ± 4.15	25.5 ± 3.80	1.366	0.174
E, (m/sec)	0.70 ± 0.10	0.65 ± 0.11	2.867	0.005
E/A	0.85 ± 0.11	0.83 ± 0.17	0.863	0.38
E/e’	8.9 ± 1.88	9.1 ± 2.26	-0.821	0.41
METS	9.11 ± 1.28	8.57 ± 1.84	1.521	0.266
Stress duration, (min)	7.4 ± 1.30	7.2 ± 1.80	0.692	0.49
Stress echocardiography				
Negative	90 (95.7)	46 (82.1)	7.672	0.006
Positive	4 (4.3)	10 (17.9)		

These findings suggest that while most conventional echo parameters were similar between the groups, patients with reduced GLS were more likely to have positive stress echocardiography results, indicating a higher prevalence of inducible ischemia in this group.

Variables of stress echocardiography

All patients underwent stress echocardiography as part of the study. Of these, 83 patients (55.3%) underwent ESE, while 67 patients (44.7%) underwent DSE. The mean duration of the stress test was 7.39±1.50 minutes, indicating the time frame over which patients were able to sustain the stress protocol. All variables of stress echocardiography are summarized in Table 3.

**Table 3:** Variables included in stress echocardiography. The data are represented in mean±SD or n (%). DSE: dobutamine stress echocardiography; ESE: exercise stress echocardiography; METS: metabolic equivalents.

Variables	N=150 patients
ESE	83 (55.3)
DSE	67 (44.7)
Positive test	14 (9.3)
Duration of stress, (min)	7.39±1.50
METS	8.9±1.48

Demographics among positive and negative stress echocardiography

Among all the patients in the study, 136 had negative stress echocardiography results, while 14 had positive results. In Table 4, a comparison of demographic details among positive and negative stress echocardiography is shown. Hypertension was more prevalent in the stress echocardiography positive group (42.8% vs. 28.6%), although this difference did not reach statistical significance (p=0.27). Smoking status showed a significant difference between the groups, with a higher prevalence in the stress echocardiography-positive group (50.0% vs. 22%; p=0.02).

**Table 4:** : Comparison of demographic among positive and negative stress echocardiography. The data is represented in mean±SD or n (%). p-value <0.05 is considered statistically significant. BMI: body mass index; DM: diabetes mellitus; HbA1C: glycated hemoglobin.

Variables	Stress echo negative (N=136 patients)	Stress echo positive (N=14 patients)	t or chi-square value	p-value
BMI, (kg/m^2^)	25.14 ± 3.49	25.08 ± 4.05	0.061	0.95
Age, years	58.07 ± 12.60	63.5 ± 10.99	-1.537	0.12
Hypertension	39 (28.6)	6 (42.8)	1.215	0.27
Dyslipidemia	66 (48.5)	8 (57.1)	0.377	0.53
Smoking	30 (22)	7 (50.0)	5.333	0.02
Hypothyroidism	31 (22.8)	6 (42.8)	2.750	0.09
HbA1C	7.57	8.48	-2.556	0.012
Duration of DM	7.60	10.22	-1.575	0.11

Patients with positive stress echocardiography results showed significantly lower average GLS compared to those with negative results (16.16±3.96% vs 18.69±3.45%; p=0.04). Table 5 shows an analysis of GLS and LVEF in patients with positive versus negative stress echocardiography results.

**Table 5:** Comparison of GLS and LVEF parameters among positive and negative stress echocardiography. The data is represented in mean±SD or n (%). p-value <0.05 is considered statistically significant. GLS: global longitudinal strain; LVEF: left ventricular ejection fraction; METS: metabolic equivalents.

Variables	Stress echo negative (N=136 patients)	Stress echo positive (N=14 patients)	t or chi-square value	p-value
LVEF, %	59.8 ± 3.77	58.15 ± 3.73	1.641	0.19
Average GLS, %	18.69 ± 3.45	16.16 ± 3.96	2.083	0.04
METS	8.9 ± 1.45	7.66 ± 0.67	3.051	0.003
Stress duration, (min)	7.9 ± 1.46	5.7 ± 0.67	3.783	<0.001

## Discussion

It is well recognized that LVEF often remains within the normal range even when significant impairment in myocardial deformation has already occurred. This underscores the importance of utilizing more sensitive measures to detect early cardiac dysfunction in this population [11]. Previous studies have reported a wide range of prevalence for myocardial dysfunction in diabetic individuals, varying from 21% to 63% depending on the echocardiography techniques employed [14,15]. Our results align with this observation, as we found evidence of LV systolic dysfunction in 37.7% of the diabetic patients in our study.

The GLS was adopted as the preferential indicator to evaluate the global systolic function [7]. The present study found a significant correlation between HbA1c levels and reduced GLS, independent of conventional cardiovascular risk factors, which corresponded to the previous research [7,16]. Additionally, our study did not identify BMI as an independent risk factor, which contrasts with findings from another study [17]. This discrepancy might be attributed to selection bias, insufficient sample size, or potential inaccuracies in BMI measurements. In our study, we observed that T2DM patients with reduced GLS had a higher prevalence of hypertension compared to those with GLS≥-17%. This finding aligns with research from the Framingham Heart study, which demonstrated significant combined effects of hypertension and diabetes on GLS [18]. To our knowledge, while numerous studies have demonstrated LV systolic dysfunction using GLS in patients with diabetes, relatively few have combined GLS with stress echocardiography to assess LV systolic dysfunction in diabetic patients with preserved ejection fraction [8,11,19,20]. Among these, Liu et al. [20] investigated whether GLS provides prognostic value in these patients and found that impaired GLS was associated with cardiovascular events and offered incremental prognostic value in T2DM patients. Additionally, Duan et al. assessed LV reserve function using stress echocardiography in asymptomatic T2DM patients and concluded that combining stress echocardiography with GLS might be valuable in detecting subtle myocardial injury in this population [8].

The literature suggests that GLS and stress echocardiography offer superior sensitivity compared to LVEF in evaluating ventricular function. These techniques have proven particularly valuable in characterizing cardiac changes in patients with diabetes, often revealing subtle alterations even in the early stages of the disease. This enhanced sensitivity allows for earlier detection of myocardial dysfunction [8]. Similarly, in the present study, we examined the relationship between stress echocardiography and GLS. The findings reveal a correlation between these two parameters. In particular, 10 patients exhibited inducible ischemia accompanied by reduced GLS, suggesting LV systolic dysfunction. It underscores the potential limitations of relying on a single diagnostic modality and emphasizes the correlation between LV systolic dysfunction and inducible ischemia in patients with diabetes. This combined approach may lead to more accurate diagnosis in patients with diabetes. Furthermore, when analyzing the average GLS values across patient groups based on their stress echocardiography outcome, we observed patients with positive stress echocardiography demonstrated reduced GLS values.

Our study has several limitations. The single-center design and relatively small sample size of 150 patients may limit the generalizability of our findings. The cross-sectional nature prevents conclusions about causality or the long-term prognostic value of reduced GLS. The use of both exercise and dobutamine stress echocardiography protocols may introduce variability in ischemia detection. Further large-scale, longitudinal studies are needed to validate these findings and explore their clinical implications. An important limitation to note is the potential for referral bias in our study population, as we included only patients who were referred for stress echocardiography. This selection criterion may have resulted in a study cohort that differs from the general population of T2DM patients with preserved LVEF. Patients referred for stress echocardiography might have had specific clinical indications or risk factors that prompted their referral, potentially leading to an overestimation of the prevalence of LV dysfunction and positive stress test results compared to the broader diabetic population. Future studies should consider including a more diverse sample of diabetic patients, regardless of their referral status for stress testing, to better represent the general T2DM population.

## Conclusions

This study demonstrates that asymptomatic T2DM patients with preserved ejection fraction can exhibit LV systolic dysfunction, detectable through reduced GLS values. These lower GLS values may represent the earliest indication of diabetic heart disease, showing strong correlations with the duration of diabetes and HbA1c levels. This finding is particularly noteworthy as it challenges traditional diagnostic approaches that rely solely on ejection fraction measurements. Importantly, impaired LV-GLS was independently associated with positive stress echocardiography results, suggesting a link between subclinical dysfunction and silent ischemia. These findings underscore the importance of advanced echocardiographic techniques in the early detection of cardiac changes in diabetic patients, potentially allowing for more proactive cardiovascular management and intervention before the development of overt heart disease.

## References

[1] Urlic H, Kumric M, Vrdoljak J (2023). Role of echocardiography in diabetic cardiomyopathy: from mechanisms to clinical practice. J Cardiovasc Dev Dis.

[2] Katogiannis K, Vlastos D, Kousathana F (2020). Echocardiography, an indispensable tool for the management of diabetics, with or without coronary artery disease, in clinical practice. Medicina (Kaunas).

[3] Stratton IM, Adler AI, Neil HA (2000). Association of glycaemia with macrovascular and microvascular complications of type 2 diabetes (UKPDS 35): prospective observational study. BMJ.

[4] Mochizuki Y, Tanaka H, Matsumoto K (2015). Clinical features of subclinical left ventricular systolic dysfunction in patients with diabetes mellitus. Cardiovasc Diabetol.

[5] Kumar A, Gupta S (2021). Evaluation of cardiac systolic function in asymptomatic diabetes patients and its correlation with duration of diabetes. Paripex.

[6] Yamauchi Y, Tanaka H, Yokota S (2021). Effect of heart rate on left ventricular longitudinal myocardial function in type 2 diabetes mellitus. Cardiovasc Diabetol.

[7] Chen Y, Zhang Y, Wang Y (2023). Assessment of subclinical left ventricular systolic dysfunction in patients with type 2 diabetes: relationship with HbA1c and microvascular complications. J Diabetes.

[8] Duan Y, Ye L, Shu Q (2023). Abnormal left ventricular systolic reserve function detected by treadmill exercise stress echocardiography in asymptomatic type 2 diabetes. Front Cardiovasc Med.

[9] Ilardi F, Santoro C, Maréchal P (2021). Accuracy of global and regional longitudinal strain at peak of dobutamine stress echocardiography to detect significant coronary artery disease. Int J Cardiovasc Imaging.

[10] Yang H, Wright L, Negishi T, Negishi K, Liu J, Marwick TH (2018). Research to practice: assessment of left ventricular global longitudinal strain for surveillance of cancer chemotherapeutic-related cardiac dysfunction. JACC Cardiovasc Imaging.

[11] Ng AC, Bertini M, Ewe SH, van der Velde ET, Leung DY, Delgado V, Bax JJ (2019). Defining subclinical myocardial dysfunction and implications for patients with diabetes mellitus and preserved ejection fraction. Am J Cardiol.

[12] Bansal M, Kasliwal RR (2013). How do I do it? Speckle-tracking echocardiography. Indian Heart J.

[13] Pellikka PA, Nagueh SF, Elhendy AA, Kuehl CA, Sawada SG (2007). American Society of Echocardiography recommendations for performance, interpretation, and application of stress echocardiography. J Am Soc Echocardiogr.

[14] Boyer JK, Thanigaraj S, Schechtman KB, Pérez JE (2004). Prevalence of ventricular diastolic dysfunction in asymptomatic, normotensive patients with diabetes mellitus. Am J Cardiol.

[15] Fang ZY, Schull-Meade R, Downey M, Prins J, Marwick TH (2005). Determinants of subclinical diabetic heart disease. Diabetologia.

[16] Silverii GA, Toncelli L, Casatori L, Bossini R, Nannelli F, Pala L, Mannucci E (2022). Assessment of left ventricular global longitudinal strain in patients with type 2 diabetes: relationship with microvascular damage and glycemic control. Nutr Metab Cardiovasc Dis.

[17] Tseng CH (2006). Body mass index and waist circumference as determinants of coronary artery disease in Taiwanese adults with type 2 diabetes mellitus. Int J Obes (Lond).

[18] von Jeinsen B, Vasan RS, McManus DD, Mitchell GF, Cheng S, Xanthakis V (2020). Joint influences of obesity, diabetes, and hypertension on indices of ventricular remodeling: findings from the community-based Framingham Heart Study. PLoS One.

[19] Holland DJ, Marwick TH, Haluska BA (2015). Subclinical LV dysfunction and 10-year outcomes in type 2 diabetes mellitus. Heart.

[20] Liu JH, Chen Y, Yuen M (2016). Incremental prognostic value of global longitudinal strain in patients with type 2 diabetes mellitus. Cardiovasc Diabetol.

